# Longer time to peak glucose during the oral glucose tolerance test increases cardiovascular risk score and diabetes prevalence

**DOI:** 10.1371/journal.pone.0189047

**Published:** 2017-12-07

**Authors:** Yi-Chun Lin, Harn-Shen Chen

**Affiliations:** 1 Division of Endocrinology and Metabolism, Department of Medicine, Taipei Veterans General Hospital, Taipei, Taiwan; 2 Faculty of Medicine, National Yang-Ming University School of Medicine, Taipei, Taiwan; Florida International University Herbert Wertheim College of Medicine, UNITED STATES

## Abstract

**Introduction:**

The pattern of glucose levels during an oral glucose tolerance test (OGTT) may be useful for predicting diabetes or cardiovascular disease (CVD). Our aim was to determine whether the time to peak glucose during the OGTT is associated with CVD risk scores and diabetes.

**Methods:**

Individuals with impaired fasting glucose (IFG) were enrolled in this observational study. Participants were grouped by the measured time to peak glucose (30, 60, 90 and 120 min) during the 75g OGTT. The primary outcome was 10-year CVD risk scores (using the Framingham risk score calculator). Secondary outcomes evaluated effect of time to peak glucose on prevalence of diabetes and indicators of glucose homeostasis.

**Results:**

A total of 125 patients with IFG underwent OGTTs. Framingham 10-year risk score for the 90-min group was 1.7 times higher than for the 60-min group (6.98±6.56% vs. 4.05±4.60%, P = 0.023). Based on multivariate linear regression, time to peak glucose at 90 min was associated with a higher Framingham risk score than 60-min group (β coefficient: 2.043, 95% confidence interval: 0.067–6.008, P = 0.045). The percentages of patients with HbA_1c_ ≥6.5%, isolated post-challenge hyperglycemia (IPH) and diabetes (combined IPH and HbA_1c_ ≥6.5%) were significantly increased with longer times to peak glucose. Prevalence of diabetes was higher in the 90-min group than in the 60-min group (31.5% vs. 5.7%, P = 0.001).

**Conclusions:**

In **s**ubjects with IFG, those with a longer time to peak glucose had a higher Framingham 10-year risk score and were associated with a greater likelihood of IPH and diabetes.

## Introduction

The 2-h 75g oral glucose tolerance test (OGTT) has been used for diagnosis of diabetes. In addition to fasting and 2-h post-load glucose levels, which are used in the diagnosis of diabetes, multiple metabolic indices can be obtained from OGTTs. Some of these might provide insights for defining risk of diabetes in the future. Such indices include time to peak glucose [1], shape of the glucose curve [2], relationship between post-load glucose and fasting glucose level [3], and insulin concentration pattern [4]. Previous studies concluded that delayed nadir for post-load glucose level indicated a higher insulin resistance combined with a reduction in insulin secretion–leading to a higher risk of diabetes. In patients with impaired glucose tolerance (IGT), there is a delay in peak insulin response during the OGTT relative to normal subjects [5–7]. OGTTs also revealed an attenuated insulin response in subjects with IGT who later developed type 2 diabetes [8,9]. A major drawback, however, is that the results of OGTTs reflect a complex interplay between insulin secretion and insulin resistance, and this may affect the reproducibility of the results. In a Canadian study, where OGTTs were repeated three times in healthy adults, time to peak glucose had the highest reproducibility, and was delayed in early type 2 diabetes [10]. However, whether time to peak glucose corresponds to increased cardiovascular risk is unknown. The aim of the present study was to determine if cardiovascular risk is associated with time to peak glucose during the OGTT in individuals with impaired fasting glucose. We also investigated the effects of time to peak glucose on prevalence of diabetes and indices of glucose homeostasis.

## Materials and methods

### Subjects

The participants analyzed in this study were from the original study of “The influence of statins on glucose homeostasis and the biomarkers of diabetes in subjects with impaired fasting glucose” which was conducted to evaluate the risk and possible mechanism of statin related diabetes. From January, 2011 to December, 2014, a total of 125 study subjects with impaired fasting glucose (IFG) were enrolled from outpatient department of a medical center and then randomized to receive rosuvastatin, pravastatin or placebo to evaluate the effects on the glucose homeostasis and other biomarkers. Impaired fasting glucose (IFG), defined as fasting plasma glucose between 100 and 125 mg/dL, is a type of pre-diabetes and an intermediate state in the transition of glucose tolerance from normal to diabetes. We use the baseline data of this cohort to compare the OGTT pattern and cardiovascular risk score as well as glucose homeostasis indicators. There were no drop-out patients. Exclusion criteria included diabetes, use of anti-diabetic medications, serum creatinine >1.4 mg/dL, alanine aminotransferase (ALT) >120 U /L, presence of active malignancy, and other endocrine disorders which can influence glucose homeostasis. The study was approved by the Institutional Review Board of the Taipei Veterans General Hospital, and written informed consent was obtained from all participants.

### Study design

For OGTTs, participants consumed 75 g of glucose (dissolved in 300 mL of water) after an 8-h overnight fast. An intravenous catheter was inserted near the antecubital fossa, to draw blood. Blood samples were obtained during the OGTT at 0, 30, 60, 90 and 120 min, in order to measure insulin and glucose levels at each of these time points. Body mass index (BMI) was calculated as weight (kg) divided by height (m) squared. Blood pressure was recorded in the sitting position using an automated sphygmomanometer as the average of two readings.

### Measurements

Insulin was measured using the Immulite immunoassay (Diagnostic Products Corporation, Los Angeles, CA, USA). The intra-assay coefficient of variation (CV) is 5.3%, and the inter-assay CV is 6.1%. The analytical sensitivity is 2 μIU/mL. Serum glucose was measured by the glucose oxidase method in a glucose analyzer (model 2300, YSI Incorporated, Yellow Springs, OH, USA). HbA_1c_ was measured by high-performance liquid chromatography (HLC-723G7, Tosoh, Japan) with a reference range of 4.2–5.8%.

Areas under the curve (AUC) for glucose and insulin during the OGTT was calculated by the trapezoid rule. Early-phase insulin secretion (insulinogenic index) was calculated as the ratio between the incremental plasma insulin and glucose concentrations during the first 30 min of the OGTT (ΔInsulin_0–30_/ΔGlucose_0–30_). Total insulin secretion was calculated as the ratio between the incremental areas under the insulin and glucose curves during the OGTT [ΔInsulin_AUC_/ΔGlucose_AUC_]. The Matsuda index was calculated for insulin resistance as previously reported [11]. Homeostatic model assessment was used to estimate insulin resistance (HOMA-IR) and β-cell function (HOMA-β) [12]. Isolated post-challenge hyperglycemia (IPH) was defined as 2-h glucose ≥200 mg/dL during the OGTT with fasting glucose <126 mg/dL. Diabetes was diagnosed as combined IPH and HbA_1c_ ≥6.5%.

### Clinical outcome

Patients were grouped based on whether time to peak glucose occurred at 30, 60, 90 or 120 min (Group 30 min, Group 60 min, Group 90 min and Group 120 min separately). The primary outcome of this study was Framingham 10-year risk score, calculated for each subject using the Framingham risk score calculator [13,14]. The secondary outcomes included prevalence of hyperglycemia–based on percentage of patients with HbA_1c_ ≥6.5%, IPH and confirmed diabetes–and glucose homeostasis which was studied using indicators of insulin secretion and insulin resistance.

### Statistical analysis

Statistical analysis was performed using the Statistical Package for Social Sciences (SPSS) software (Version 18.0, SPSS Inc., Chicago, Illinois, USA). All data are expressed as mean ± standard deviation or frequency (percentage). Parametric continuous data between different groups were compared using the unpaired Student t‐test, and non-parametric data using the Mann‐Whitney U test. Categorical data between different groups were compared using the Chi‐squared test with Yates’ correction or Fisher’s exact test, where appropriate. Univariable linear regression was performed to evaluate factors associated with Framingham risk score. Factors with a P value <0.05 were selected for stepwise adjustment in multivariable linear regression analyses. β coefficients (95% confidence interval [CI]) were calculated, based on comparisons between different time to peak glucose groups (90 min vs. 60 min, which represented most patients) to determine if time to peak glucose was associated with Framingham risk score. Two‐sided P values <0.05 were used to infer statistical significance.

## Results

### Participant characteristics

A total of 125 subjects with IFG were recruited for this study. Time to peak glucose levels during the OGTT occurred at 30 min for four individuals, 60 min for 54 individuals, 90 min for 55 individuals, and 120 min for 9 individuals. Table 1 shows the baseline characteristics of the study participants, grouped by time to peak glucose during the OGTT. Overall, fasting plasma glucose and HbA_1c_ levels progressively increased with longer times to peak glucose (ANOVA P<0.001). Because 87% of patients experienced time to peak glucose at 60 or 90 min, we performed further analysis on these two groups. Compared with the 60-min group, individuals in the 90-min group were older (60.6±10.0 vs. 56.2±10.1 years, P = 0.023), had higher HbA_1c_ values (6.30±0.59% [45±7 mmol/mol] vs. 6.03±0.44% [42±5 mmol/mol], P = 0.009), and lower low-density lipoprotein cholesterol (LDL-C) (116.6±35.7 vs. 135.4±44.3 mg/dL, P = 0.051) (Table 1).

**Table 1:** Baseline characteristics of patients grouped by time to peak glucose level during OGTT.

	Group 30 min	Group 60 min	Group 90 min	Group 120 min	ANOVA P
n	7	54	55	9	
Male (%)	4 (57%)	14 (26%)	19 (35%)	1 (11%)	0.179
Age (years)(range)	58±12.60(39–78)	56.15±10.12(31–75)	60.58±10.02^a^(29–76)	57.33±8.12(37–79)	0.154
BMI (kg/m^2^)	24.97±7.30	24.96±3.89	24.32±2.79	25.01±3.55	0.831
SBP (mmHg)	131.86±18.91	129.14±15.67	128.96±14.46	124.89±10.52	0.819
DBP (mmHg)	78.14±16.79	77.37±9.92	76.32±9.29	75.78±7.73	0.917
FPG (mg/dL)	109.00±12.18	105.20±9.08	111.62±13.60^a^	129.67±44.32	<0.001
HbA_1c_ (%)	5.79±0.28	6.03±0.44	6.30±0.59^a^	7.2±1.72	<0.001^c^
HbA_1c_ (mmol/mol)	40±4	42±5	45±7	55±18	<0.001^c^
TC (mg/dL)	205.14±40.88	207.37±46.19	208.80±80.28	202.75±39.38	0.994
HDL-C (mg/dL)	64±35.54	51.55±10.263	51.68±13.44	54±11.53	0.333
LDL-C (mg/dL)	102.00±28.62	135.40±44.30	116.61±35.68^b^	107.57±18.53	0.094
TG (mg/dL) (median, IQR)	96	115 (80–159)	105 (88.8–226)	118 (74.3–220.5)	0.611
Creatinine (mg/dL)	0.79±0.21	0.71±0.19	0.71±0.19	0.61±0.12	0.737
ALT (U/L)	24±5.20	26.05±17.10	28.92±18.45	31.71±19.75	0.801
UACR (median, IQR)	4.2	8.5 (5.1–22.4)	7.4 (4.5–24.8)	5.7 (4.6–116.0)	0.927

Abbreviations: ALT, alanine aminotransferase; BMI, body mass index; DBP, diastolic blood pressure; FPG, fasting plasma glucose; HbA_1c_, hemoglobin A_1c_; HDL-C, high density lipoprotein cholesterol; IQR, interquartile range; LDL-C, low density lipoprotein cholesterol; SBP, systolic blood pressure; TC, total cholesterol; TG, triglyceride; UACR, urine albumin creatinine ratio

90-min group vs. 60-min group

^a^ P<0.05

^b^ P = 0.051

Trend

^c^ P = 0.08

### The Framingham 10-year risk score in groups by time to peak glucose

**Fig 1:**
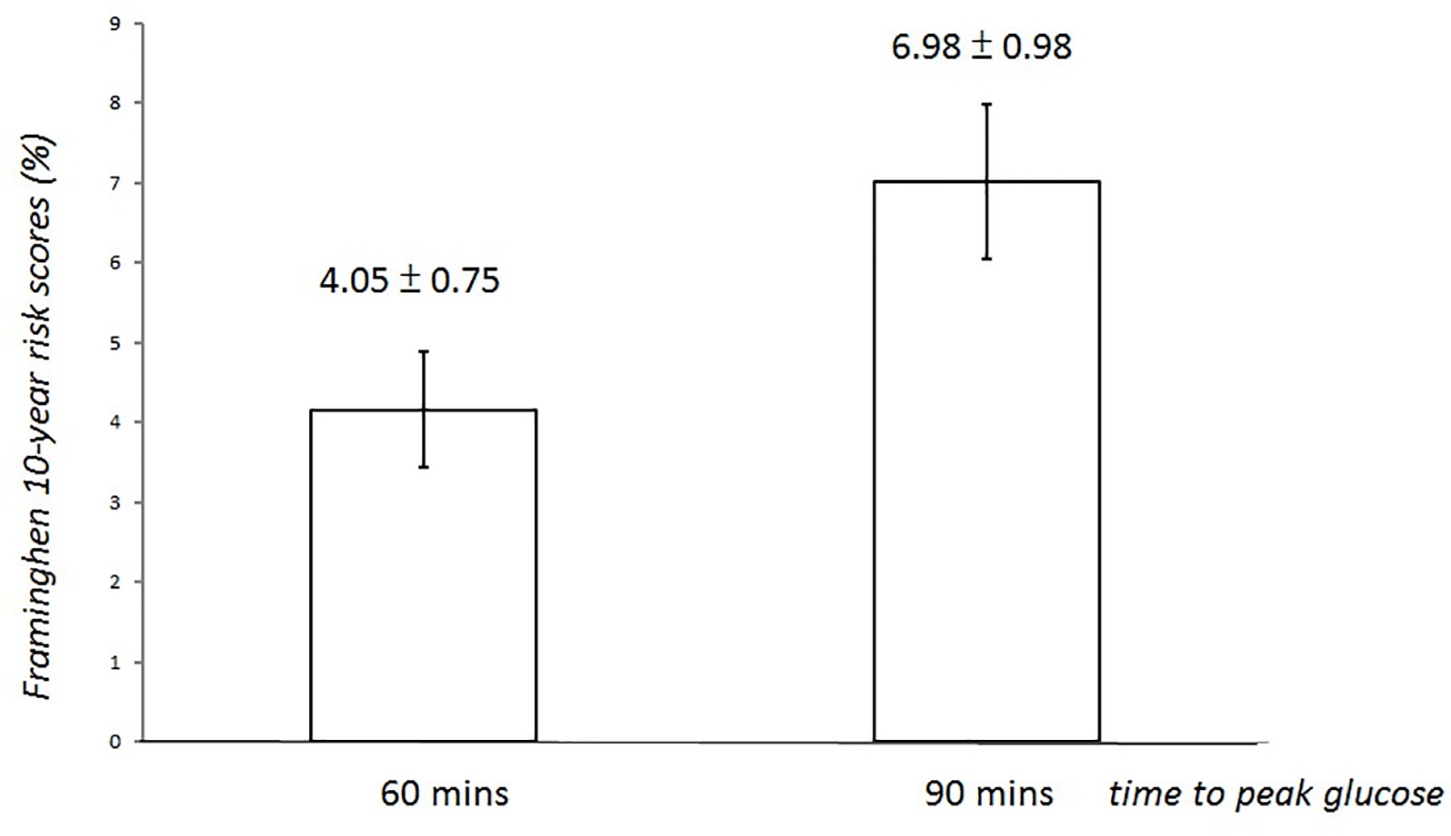
Framingham 10-year risk score of group 60-min and group 90-min.

The numbers of participants in Group 30 min and Group 120 min were much smaller than those of the Group 60 min and Group 90 min, thus Framingham 10-year risk score was compared in the Group 60 and Group 90 min. The Framingham 10-year risk score for the 90-min group was 1.7 times greater than for the 60-min group (mean± standard error: 6.98±0.98% vs. 4.05±0.75%, P = 0.01) (Fig 1). The relationship between time to peak glucose concentration and Framingham 10-year risk score was explored by linear regression analysis (Table 2). In the unadjusted model (model 1), the β-coefficient value was 2.287 (95% CI: 0.372–5.535, P = 0.025) for Group 90 min vs. Group 60 min. After adjustment for HbA_1c_, LDL and age, the β-coefficient was 2.043 (95% CI: 0.067–6.008, P = 0.045) for Group 90 min vs. Group 60 min.

**Table 2:** Association of time to peak glucose with Framingham 10-year risk score by linear regression.

	β-coefficient	95% CI	P value
Time to peak glucose(Group 90 min vs. Group 60 min)			
Model 1	2.287	0.372–5.535	0.025
Model 2	2.402	0.535–5.719	0.019
Model 3	2.404	0.615–6.653	0.019
Model 4	2.043	0.067–6.008	0.045

Model 1: unadjusted

Model 2: adjusted for HbA_1c_

Model 3: adjusted for HbA_1c_, LDL-C

Model 4: adjusted for HbA_1c_, LDL-C and age

Abbreviations: CI, confidence interval; HbA_1c_, hemoglobin A_1c_; LDL-C, low density lipoprotein cholesterol

### Prevalence of diabetes and indices of glucose homeostasis

The percentages of patients with HbA_1c_ ≥6.5% (48 mmol/mol), IPH and diabetes were also significantly increased with longer times to peak glucose (ANOVA P<0.01) (Table 3). The 90-min group, relative to the 60-min group, had more individuals with HbA_1c_ ≥6.5% (35.2% vs. 9.4%, P<0.01), IPH (83.6% vs. 29.6%, P<0.001), and diabetes (31.5% vs. 5.7%, P = 0.001) (Table 3).

**Table 3:** Prevalence of hyperglycemia in patients grouped by time to peak glucose level during OGTT.

	Group 30 min	Group 60 min	Group 90 min	Group 120 min	ANOVA P
n	7	54	55	9	
HbA_1c_ ≥6.5% (%)	0	9.4	35.2^a^	44.4	<0.01
IPH (%)	28.6	29.6	83.6^a^	88.9	<0.01^b^
DM (%)	0	5.7	31.5^a^	44.4	<0.01^c^

Abbreviations: DM, diabetes mellitus; IPH, isolated post-challenge hyperglycemia

90-min group vs. 60-min group

^a^ P<0.01

Trend

^b^ P = 0.05

^c^ P = 0.08

**Fig 2:**
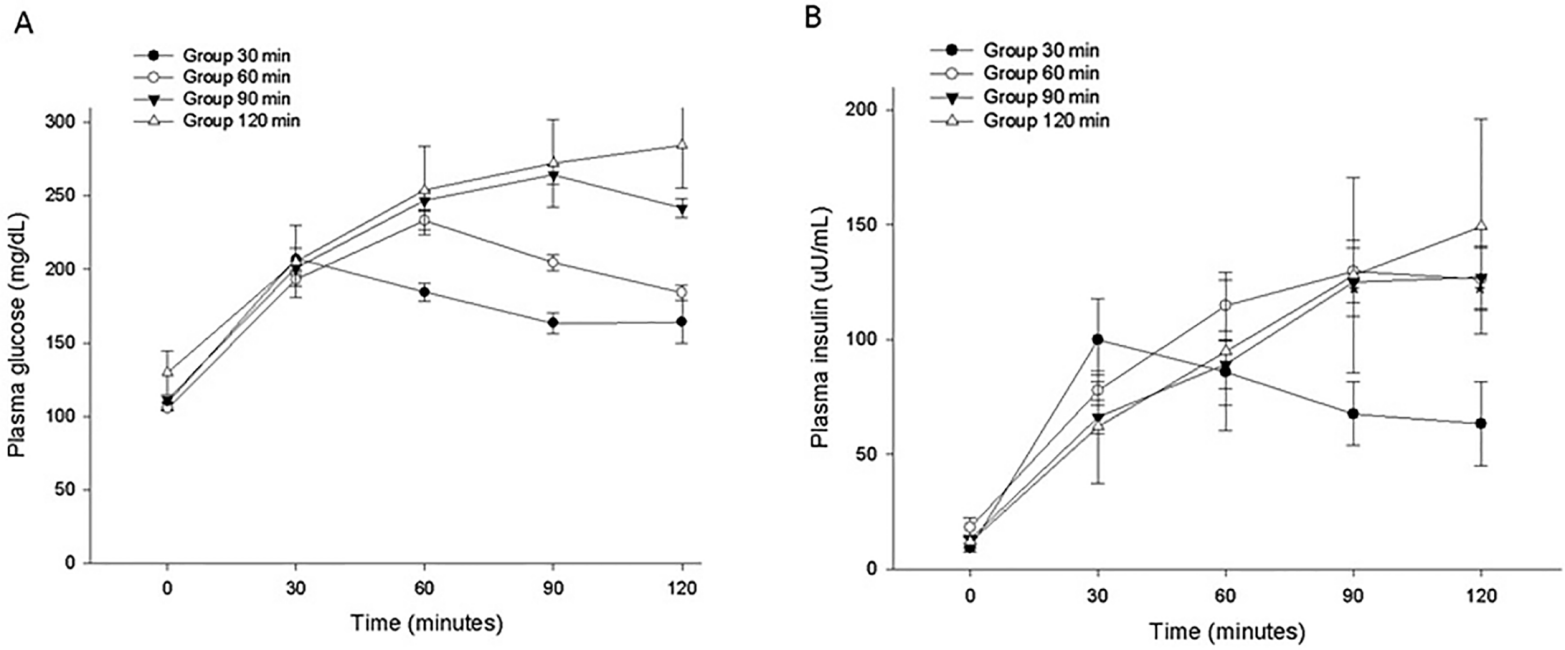
Mean ± standard error for plasma glucose (A) and insulin (B) concentration during the oral glucose tolerance test.

Mean glucose and insulin levels for each of the time to peak glucose groups are shown in Fig 2. In the 30-min group, the peak insulin level was followed by a progressive decrease, while the later insulin responses in the 60-, 90-, and 120-min groups were longer-lasting.

Due to the high prevalence of IPH and DM in Group 120 min and small sample size in Group 30 min and Group 120 min, measures of glycemia and insulin secretion during the OGTT are shown only for the Group 60 min and Group 90 min groups in Table 4. There were no significant differences between the groups for HOMA-IR, HOMA-β, insulin AUC, ΔInsulin_0-30_/ΔGlucose_0-30_ or the Matsuda index. The glucose AUC was significantly increased in the Group 90 min (389.3±65.0 vs. 44.5±74.9 mg‧h‧dL^-1^, P<0.001). However, total insulin secretion calculated by ΔInsulin_AUC_/ΔGlucose_AUC_ was higher in the Group 60 min compared with the Group 90 min, with the difference bordering on statistical significance (P = 0.052).

**Table 4:** Comparison of glycemia and insulin secretion by time to peak glucose.

	Group 60 min	Group 90 min	P value
N	54	55	
HOMA-IR	3.63±3.04	3.19±2.44	0.400
HOMA-β (%)	123.53±118.34	98.40±84.44	0.186
Glucose AUC (mg‧h‧dL^-1^)	389.29±65.00	444.50±74.90	<0.001
Insulin AUC (μU‧h‧mL^-1^)	193.62±127.89	175.25±140.28	0.486
ΔInsulin_0-30_/ΔGlucose_0-30_ (ΔU‧mg^-1^)	0.70±0.45	0.62±0.44	0.405
Matsuda index	2.67±1.60	3.14±2.31	0.477
ΔInsulin_AUC_/ΔGlucose_AUC_ (μU‧mg^-1^)	0.99±0.76	0.73±0.52	0.052

HOMA-IR = fasting glucose (mg/dL) × [fasting insulin (μU/mL)]/405. Abbreviations: AUC, area under the curve; HOMA-β, homeostasis model assessment of β-cell function; HOMA-IR, homeostasis model assessment of insulin resistance

## Discussion

Our findings showed that a longer time to peak glucose level during the OGTT of patients with IFG was associated with a higher Framingham risk score and higher prevalence of diabetes. Compared with an early time to peak glucose, individuals with a later peak had higher HbA_1c_, higher fasting plasma glucose, as well as increased glucose AUC during the OGTT with lower total insulin secretion.

Although OGTT is widely used for the diagnosis of diabetes, a major disadvantage is the difficulty in reproducing results. According to Kramer et al. [10], after performing three replicate OGTTs, the time to peak glucose was the only parameter that could be reliably reproduced. Thus, time to peak glucose during the OGTT holds the potential for use in clinical applications and future research studies.

Based on previous studies, there is evidence for associations between time to peak glucose during the OGTT and pancreatic beta cell function and insulin resistance. In a study of 2549 Chinese subjects who underwent the OGTT, those with 1-h plasma glucose >200 mg/dL were characterized by impaired early insulin secretion [15]. Even in subjects with normal glucose tolerance, subjects whose post-load plasma glucose level returned to baseline more quickly had a higher insulinogenic index and greater insulin sensitivity [3]. Our data showed that longer time to peak glucose was associated with a less favorable metabolic profile–including higher HbA_1c_ and increased fasting plasma glucose. The increase in glucose AUC during the OGTT, and the markedly higher percentage of IPH in the 90-min compared with the 60-min group, could be explained by a reduction in total insulin secretion, as insulin resistance measures (based on HOMA-IR and the Matsuda index) were similar in both groups. Although the HOMA-IR and Matsuda index had good correlation with insulin resistance index obtained from sophisticated glycemic clamp test [16,17], the primary site of insulin resistance (liver or peripheral) is not known based on current study methodology. In a previous study [4], a longer-lasting insulin response–i.e. a continuous insulin elevation sustained over 120 min–indicated increased insulin resistance and impaired early insulin response. Although longer-lasting insulin response was noted in groups with time to peak glucose longer than 30 min in our cohort, no difference was found in the present study for insulin resistance and early insulin response based on the insulinogenic index.

Insulin resistance is associated with hyperinsulinemia and leads to adverse effects on arterial endothelium [18] by several proposed mechanism including altered insulin signaling, endothelial dysfunction [18], and left ventricular diastolic function [19]. In this study, the higher Framingham 10-year risk score in the 90-min group compared with the 60-min group may be a result of hyperglycemia. Compared with fasting plasma glucose levels, 2-h plasma glucose is a better predictor for coronary heart disease [20,21] and ischemic stroke [11]. The DECODE study [22] demonstrated that individuals whose 2-h plasma glucose did not return to fasting plasma glucose levels during the OGTT had a higher risk of mortality from cardiovascular disease and greater all-cause mortality. Considering that post-challenge hyperglycemia is related to a proatherogenic profile [23], the time to peak glucose may provide additional insights for identifying individuals who are at risk of cardiovascular disease. Assessment of cardiovascular endpoints often requires a long follow-up time. In a study of normoglycemic subjects, failure of 2-h plasma glucose to return to the fasting plasma glucose level after a 75g OGTT was associated with worse cardiovascular disease profile, elevated insulin levels and increased number of cardiovascular events over a median follow-up of up to 16 years [24]. In the present study, pre-diabetic patients with a longer time to peak glucose level were associated with higher 10-year Framingham risk score. To our knowledge, this is the first study to demonstrate an association between time to peak glucose and cardiovascular risk score.

Previous studies that focused on the clinical applications of time to peak glucose have been limited. In a study that included 325 women, the time to peak glucose concentration during the OGTT, performed 3 months post-partum, was an independent predictor for future dysglycemia [1]. Based on its associations with various indicators of diabetes pathophysiology, and given its reproducibility, the time to peak glucose could serve as a predictive metabolic marker of future dysglycemia or cardiovascular disease.

The current study has some limitations. Firstly, as only patients with IFG were included, the results cannot be extended to the normal population. Nevertheless, compared with individuals with normal glucose tolerance, patients with IFG or IGT have an increased risk of death [22], emphasizing a need for studies that focus on this population. Secondly, the cross-sectional design of this study did not account for cardiovascular disease events. However, the mean follow-up time for cardiovascular endpoints is usually long and a 10-year risk score may serve as a surrogate marker.

In conclusion, using time to peak glucose as a novel independent variable for predicting cardiovascular risk, our study revealed that IFG subjects with a longer time to peak glucose had a higher Framingham 10-year risk score. Longer time to peak glucose level during an OGTT was associated with higher HbA_1c_, increased glucose AUC, reduced total insulin secretion and thus a greater likelihood of IPH and diabetes. This finding suggests that time to peak glucose during the OGTT could be a useful parameter and warrants further study.

## Supporting information

S1 File Time to peak PG remove ID. (XLSX)
